# Nipple adenoma arising from axillary accessory breast: a case report

**DOI:** 10.1186/1746-1596-7-162

**Published:** 2012-11-27

**Authors:** Yoshihiro Shioi, Shin-ichi Nakamura, Shuji Kawamura, Masako Kasami

**Affiliations:** 1 Department of Surgery, Morioka Municipal Hospital, 5-15-1 Motomiya, Morioka 020-0866, Japan; 2 Mitsubishi Chemical Medience Corporation, 4-47-30 Aoyama, Morioka, Iwate, 020-0133, Japan; 3 Department of Surgery, Iwate Prefectural Esashi Hospital, 5-23 Nishioodori, Esashi, Oshu, Iwate, 023-1103, Japan; 4 Department of Pathology, Shizuoka Cancer Hospital, 1007 Shimonagakubo, Nagaizumi, Sunto, Shizuoka, 411-8777, Japan

**Keywords:** Nipple adenoma, Accessory breast

## Abstract

Nipple adenoma is a relatively rare benign breast neoplasm, and cases of the disease arising from the axillary accessory breast have very seldom been reported in the English literature. We report a case of nipple adenoma arising from axillary accessory breast including clinical and pathological findings. An 82-year-old woman presented with the complaint of a small painful mass in the right axilla. Physical examination confirmed a well-defined eczematous crusted mass that was 8 mm in size. The diagnosis of nipple adenoma was made from an excisional specimen on the basis of characteristic histological findings. Microscopic structural features included a compact proliferation of small tubules lined by epithelial and myoepithelial cells, and the merging of glandular epithelial cells of the adenoma into squamous epithelial cells in the superficial epidermal layer. Because clinically nipple adenoma may resemble Paget’s disease and pathologically can be misinterpreted as tubular carcinoma, the correct identification of nipple adenoma is an important factor in the differential diagnosis for axillary tumor neoplasms.

**Virtual slides:**

The virtual slide(s) for this article can be found here: http://www.diagnosticpathology.diagnomx.eu/vs/1186821489769063

## Background

Nipple adenoma is a benign proliferative lesion of the breast that arises from galactophorous duct of the nipple. It was first recognized as a distinctive entity in 1955 by Jones who referred to it as “florid papillomatosis” of the nipple duct [1]. Nipple adenoma, also known as nipple duct adenoma, papillary adenoma, erosive adenomatosis, florid papillomatosis, papillomatosis of the nipple and subareolar duct papillomatosis, is a variant of intraductal papilloma involving the terminal portion of the galactophorous ducts [2-5]. Clinically, nipple adenoma can be mistaken for Paget’s disease and can be interpreted pathologically as a tubular carcinoma. Although axillary tumors have many differential diagnoses ranging from benign to malignant, nipple adenoma arising from the axillary accessory breast has rarely been described in the English literature. We describe the clinical and pathological finding relating to a rare case of nipple adenoma arising in an axillary accessory breast.

## Case presentation

**Figure 1:**
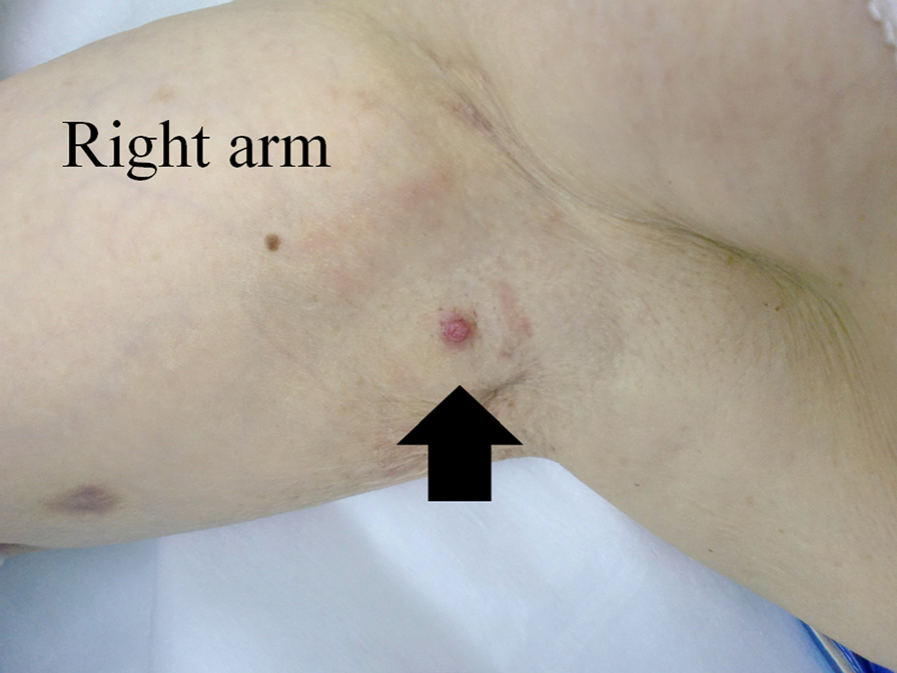
**Clinical appearance. **Photograph showing a well-circumscribed eczematous crusted tumor with erythema, 8 mm of the size, and located in the right axilla.

An 82-year-old Japanese woman presented with the complaint of a painful tumor that had been localized in the right axilla. The tumor was a well-circumscribed eczematous crusted tumor with erythema that was 8 mm in size, and exhibited erosion but no discharge (Figure 1). The tumor was separate from the patient’s breast and the axillary lymph nodes were not palpable. It was suspected as being an inflamed epidermal cyst, furuncle or possibly extramammary Paget’s disease. No neoplastic lesions were detected in the patient’s breast. Complete local excision was performed under local anesthesia. Histologically, the tumor was diagnosed as nipple adenoma arising from the axillary accessory breast. The patient has had no local recurrence at 2 years after excision of the tumor.

## Pathological findings

**Figure 2:**
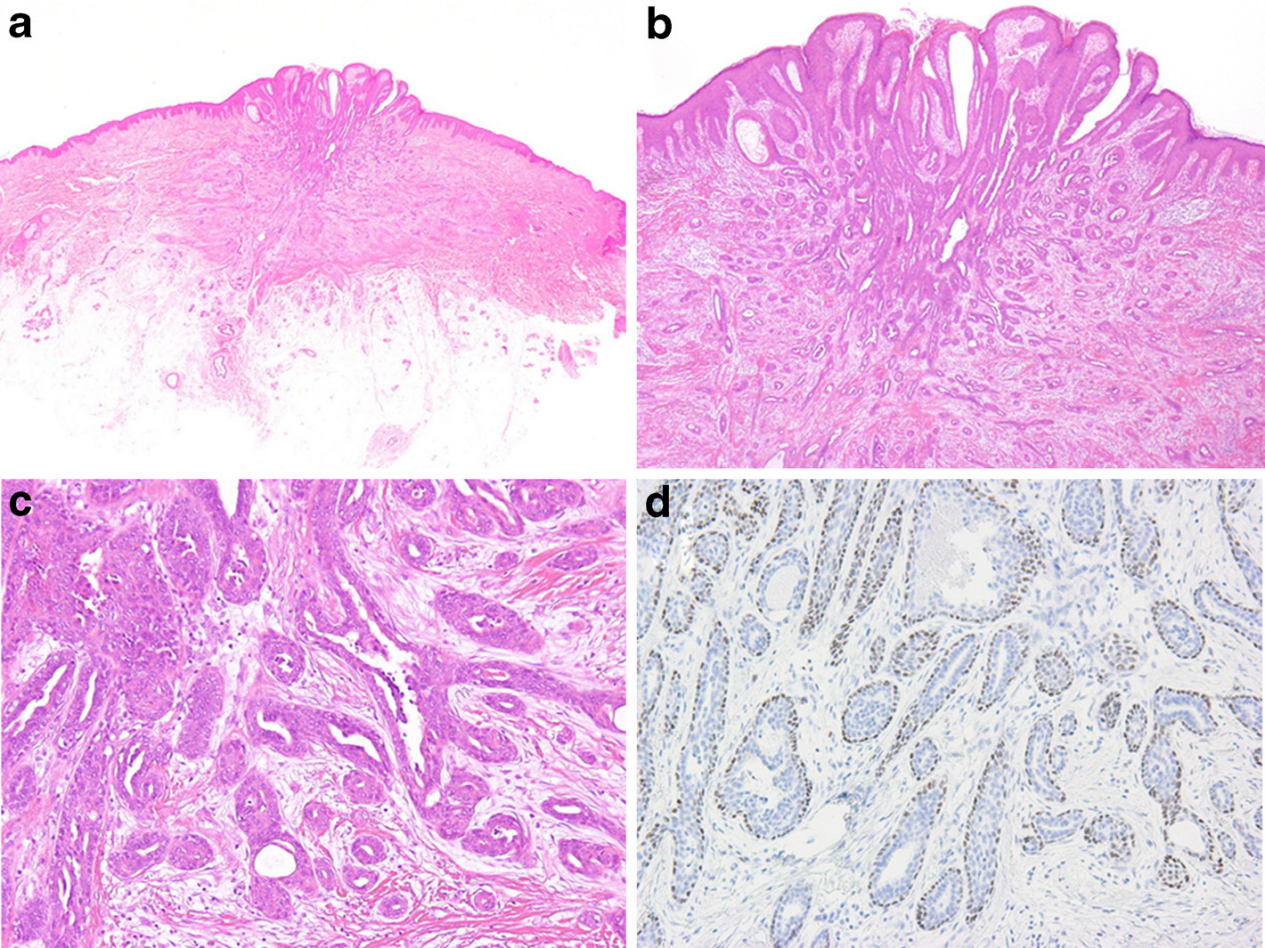
**Loupe images showing cross-sections of the tumor. a **At the top of the section the epidermis of the accessory nipple can be seen. At the bottom of the section some ducts and atrophic lobules are visible. **b** A low-power view showing the architecture with a complex proliferation of ductules around dilated lactiferous ducts. The lesion is relatively well-circumscribed but has no capsules [×20]. **c** A higher magnification view demonstrating ductules with internal micropapillary proliferations [×100]. **d** Immunohistochemical staining for p63 to assist the recognition of the two cell layers and demonstrate the participation of myoepithelial cells [×100].

In cross section the tumor was 8 mm in diameter, appeared non-encapsulated and firm, and had no continuity with normal breast tissue. Loupe images revealed that the tumor was composed of a fairly well-circumscribed but non-encapsulated mass with some ducts and atrophic mammary lobules at its bottom (Figure 2, a). Microscopically, the main morphological feature was a clearly defined proliferation of ductules around dilated lactiferous ducts. Some ductules showed micropapillary epithelial hyperplasia. They were proliferating two cell layered glands sprouting from and compressing the ducts resembled adenosis (Figure 2, b). The tumor exhibited central dilated lactiferous ducts and a pseudoinvasive tubular pattern with dense stroma in the peripheral regions (Figure 2, c). The glandular cells had fairly regular, uniform, round to oval nuclei. There was no hyperchromasia, pleomorphism or mitotic activity. Inflammatory cell infiltration was mild around the ductules, and fibrosis was considerable. Immunohistochemical studies using the p63 stain clearly demonstrated a two layer structure composed of an epithelial layer and a myoepithelial layer (Figure 2, d). Paget’s cells were not identified in the epidermis.

## Discussion

Accessory breast tissue generally develops along the embryonic mammary ridge that extends from the axilla to the groin and is most common in the axilla. Accessory axillary breast tissue has a relatively common occurrence with an incidence of 0.4-6% [6,7]; it is one of the factors used in the differential diagnosis of axillary tumors. A number of different neoplasms, both benign and malignant, have been found in axillary breast tissue. The most common of these tumors reported in the literature is a fibroadenoma, and there are scattered case reports of other tumors including phyllodes tumor and mammary carcinoma [8].

Nipple adenoma is one of the rare benign breast tumor types which develop within or in the superficial portion of the nipple. On physical examination, the most common findings are an eroded, ulcerated, crusted nipple and a palpable nodule [3]; and nipple adenoma can be mistaken clinically for Paget’s disease [9]. In the present case, the tumor arose from the axillary accessory breast and not the nipple, and extramammary Paget’s disease was hard to diagnose preoperatively. The tumor was small, eczematous and crusted, and was initially suspected as being an inflammatory disease such as an inflamed epidermal cyst. The present case is only the 3^rd^ case of nipple adenoma arising from axillary accessory breast to be reported in the English literature, having been reported by Doctor and Shinn [5,10].

Microscopically, nipple adenoma is composed of a proliferation of small tubular structures displaying double layers [11]. Nipple adenoma is a complex benign mammary proliferation that has a variety of histologic appearances. Rosen and Caicco classified nipple adenoma into four morphological patterns: 1) sclerosing papillomatosis; 2) papillomatosis; 3) adenosis; and 4) mixtures of these proliferative patterns [4]. In our case, the tubular structure formed a complex branching pattern with some micropapillary epithelial hyperplasia and was classified as having a mixed pattern. The galactophorous ducts had a squamous cell metaplasia close to the skin. This feature is also a valuable criterion for the identification of nipple adenoma [1,2]. An adenosis and pseudoinfiltrative pattern were also prominent in the present case, and resembled invasive tubular carcinoma or adenosquamous carcinoma. However, demonstration of the two layer structure consisting of a myoepithelial layer surrounding the epithelial tubules, and the relative uniformity and coherence of the cells, indicated the benign nature of the tumor [2]. Immunohistochemical staining for p63 was particularly helpful in confirming the two layered structure (Figure 2, d). Syringomatous adenoma can be excluded as a diagnosis by the absence of irregular, compressed or comma-shaped nest infiltration into smooth muscle bundles, showing sweat gland differentiation [12].

Standard treatment for nipple adenoma is local excision [3]. Although nipple adenoma has basically been suggested as being a benign tumor, the relationship between nipple adenoma and carcinoma has not been elucidated entirely [4]. This is necessary to confirm the requirement for complete resection of the tumor and pathological retrieval. Recognition of this disease by both the clinician and the pathologist, and close communication between them is important in avoiding misdiagnosis of malignancy and unnecessarily extensive surgery.

## Conclusions

A case of nipple adenoma arising from axillary accessory breast was reported here. The correct identification of nipple adenoma is an important factor in the differential diagnosis for axillary tumors. Recognition of this rare benign condition is important in preventing misdiagnosis of malignancy.

## Consent

Written informed consent was obtained from the patient for publication of this Case report and any accompanying images. A copy of the written consent is available for review by the Editor-in-Chief of this journal.

## Competing interests

The authors declare that they have no competing interests.

## Authors’ contributions

YS and SK treated this patient clinically. S-iN, and MK contributed to pathological diagnosis. YS and MK participated in the sequence alignment and drafted the manuscript. All authors read and approved the final manuscript.
